# Nonverbal Executive Function is Mediated by Language: A Study of Deaf and Hearing Children

**DOI:** 10.1111/cdev.12659

**Published:** 2016-11-10

**Authors:** Nicola Botting, Anna Jones, Chloe Marshall, Tanya Denmark, Joanna Atkinson, Gary Morgan

**Affiliations:** ^1^ City University of London; ^2^ University College London

## Abstract

Studies have suggested that language and executive function (EF) are strongly associated. Indeed, the two are difficult to separate, and it is particularly difficult to determine whether one skill is more dependent on the other. Deafness provides a unique opportunity to disentangle these skills because in this case, language difficulties have a sensory not cognitive basis. In this study, deaf (*n* = 108) and hearing (*n* = 125) children (age 8 years) were assessed on language and a wide range of nonverbal EF tasks. Deaf children performed significantly less well on EF tasks, even controlling for nonverbal intelligence and speed of processing. Language mediated EF skill, but the reverse pattern was not evident. Findings suggest that language is key to EF performance rather than vice versa.

The association of executive function (EF) and language is one that is currently of fast growing interest to researchers and clinicians. EF is a term used to define the complex set of cognitive abilities which enable us to coordinate mental processes and manipulate information, solve novel problems, sequence information, and generate new strategies to accomplish goals in a flexible way (Elliott, 2003; Funahashi, 2001). In addition to storing information in short‐term memory, children also need to be able to process information flexibly, inhibit nonuseful responses, and manage the input in order to achieve success on higher level cognitive tasks. In the Baddeley (2003) model of working memory, EFs are served by short‐term phonological and visuospatial systems. EF development has been linked with several important associated domains, in particular, behavioral self‐regulation and social‐emotional competence (McClelland, Cameron, Wanless, & Murray, 2007). Additionally, the impact of environmental factors, such as childhood poverty, indicates EF is malleable, especially in early childhood (Raver, Blair, & Willoughby, 2013).

Language is also a key developmental skill and it is well established that EF and language are highly interrelated. Work focusing on children with typical development confirms this association (see Kuhn, Willoughby, Wilbourn, Vernon‐Feagans, & Blair, 2014 for a large prospective study), as does evidence from atypical groups such as those with developmental language impairment (Henry, Messer, & Nash, 2012) and autism (Akbar, Loomis, & Paul, 2013).

It has been difficult to untangle the direction of influence in previous research. Some theories argue that language development is more important for EF abilities than the other way around. Zelazo et al.'s cognitive complexity and control theory (Zelazo & Frye, 1998; Zelazo et al., 2003), for example, argues that rules derived from language learning enable manipulation of cognitive processes via internal representations. In the few existing longitudinal studies, early language appears to predict later self‐regulation skills and EF in typically developing children (Kuhn et al., 2014; Petersen, Bates, & Staples, 2015) more than the other way around. However, other schools of thought posit cognitive development, including executive memory skills, as a necessary component of language development in typical (Baddeley, 2003) and atypical (Pellicano, 2010) groups. Although language and EF are likely to be at least partially bidirectionally related during development, a model that identifies which is the stronger influence would be useful both theoretically and clinically.

One investigative approach that attempts to address this question is the comparison of typically developing children with individuals who have developmental disorders. Such populations present an opportunity to explore development when particular skills are less than optimal. They therefore offer the potential to discover more about the direction of relations between different developmental skills that are not as visible when development is proceeding as expected. It is already well established, for example, that children with developmental disorders such as autism (Hughes, Russell, & Robbins, 1994; Ozonoff, Pennington, & Rogers, 1991), attention deficit hyperactivity disorder (Gau & Shang, 2010; Geurts et al., 2004; Pellicano, 2010; Willcutt, Doyle, Nigg, Faraone, & Pennington, 2005), those at risk of dyslexia (Gooch, Thompson, Nash, Snowling, & Hulme, 2016), and those with developmental language impairment (Henry et al., 2012; Im‐Bolter, Johnson, & Pascual‐Leone, 2006) have poorer EF than their typical peers.

Research involving children with developmental disorders (rather than reduced sensory input as in deafness) goes someway to indicating that deficits in language and EF are associated. However, when considering whether one skill mediates the other, these designs are confounded by the potential cognitive difficulties seen in these populations. In Henry et al.'s (2012) study of children with language impairment, EF was found to relate directly to language skill, but at the same time EF showed unique predictive power for membership of the clinical developmental language group, even after controlling for verbal IQ. The authors suggested that underlying cognitive difficulties may be at least partly influential in language development rather than vice versa. However, it is not clear from studies of children with developmental disorder whether EF deficits lead to poor language or whether other cognitive factors are also at play, influencing the development of both EF and language development (Bishop, Nation, & Patterson, 2014).

In contrast, children who are deaf but otherwise typically developing are an interesting group to study in this respect. Deaf children offer a relatively “pure” way of exploring the association of EF and language because their language development is delayed by sensory factors rather than by a cognitive deficit per se. The majority of deaf children have normal cognitive ability, as measured by nonverbal IQ tasks, in contrast to their delayed language skills (Marschark & Hauser, 2008). Nevertheless, deaf children as a group have previously been shown to perform more poorly on EF tasks (Beer, Kronenberger, & Pisoni, 2011; Figueras, Edwards, & Langdon, 2008; Hintermair, 2013; Kronenberger, Colson, Henning, & Pisoni, 2014) as well as having low scores on language tasks.

Thus far, the number of studies on deaf children that report both EF *and* language data is very small. In those that exist, methodological limitations make it difficult to reach conclusions. For example, some studies have only used questionnaire data (Hintermair, 2013) and other studies have used a limited set of only one or two experimental tasks (e.g., Oberg & Lukomski, 2011). Some research has selected only certain groups of deaf children such as those with cochlear implants (e.g., Kronenberger et al., 2014) or with hearing aids (Stiles, McGregor, & Bentler, 2012). The hearing status of the parents is also sometimes not included despite affecting performance on language tasks, with children of deaf parents scoring better (e.g., Lederberg, 2006). In most of these articles, sample size is limited, restricting the use of complex analyses on the data sets. One exception is a study by Figueras et al. (2008), which used a larger sample of deaf children and a more extensive task battery than most and which came to the conclusion that language might mediate EF. However, their study still did not have a large enough number of participants to demonstrate this statistically.

Another key difficulty with EF studies in deafness (and more generally in atypical populations) is the nature of the EF tasks themselves. In particular, two aspects are often overlooked: (a) the degree to which speed of processing is involved in completing the tasks—better performance on EF tasks might be entirely down to a simpler scanning and responding process rather than due to difficulties manipulating information and (b) the degree of verbal content present in the tasks administered, either in the explicit response required or the implicit language demands. These factors are rarely controlled for but are both likely to make to an important contribution to differences between groups. This is especially true in groups where language difficulties are known to occur (Botting, Psarou, Caplin, & Nevin, 2013). Therefore, the question of whether EF mediates language development or whether language influences EF development remains open.

## Present Study

This study investigates EF and language in typically developing children and in children who are deaf (and are at risk of language delay caused by sensory difficulties). This is important because it separates out the confounding cognitive issues seen in studies of other atypical groups. We overcome many previous methodological limitations evident in existing literature by (a) reporting on a large group of deaf children selected to be widely representative of the whole population (*n* = 108), (b) including a carefully matched comparison group of hearing peers (*n* = 125), (c) using measures carefully designed to be as explicitly and implicit nonverbal as possible and modality fair (across spoken and signed language), and (d) controlling for speed of processing and general nonverbal ability. The analyses aim to address the following questions:

1. Does atypical language *experience* (in this case being deaf) affect performance on nonverbal EF tasks compared to age‐matched hearing peers? Is this true even after controlling for general cognitive ability and speed of processing?
2. Does language correlate with EF tasks in each group?
3. Does language mediate EF differences between the groups or vice versa?

## Method

### Participants

Children from two groups were recruited, those who were deaf and those with typical hearing. All children were living in the United Kingdom or Ireland and had English or British Sign Language (BSL) as their primary language. Children with explicit additional diagnoses such as global intellectual disability, autism, cerebral palsy, or Down syndrome were not recruited to the study.

In total, 108 deaf children took part, 49 (45%) girls and 59 (55%) boys with a mean age of 8;10 years (*SD* = 1;9; range = 5;9–11;8). Most of these groups were White British (72%) with 4 of the remaining participants being mixed race, 17 Asian, and 9 from other backgrounds. Overall, 86 (84%) of these children were born deaf, and all deaf children were deaf before starting school at the age of 5. Forty‐six (45%) of the families who responded to the questionnaire reported a genetic basis to the child's deafness, 9 (9%) reported an illness‐based cause, and for 48 (47%) the cause was unknown. Nineteen deaf children were born prematurely. Twenty‐four (22%) children had deaf parents, and 16 of these 24 also had a deaf sibling. In terms of hearing level, 13 children were classed as mild/moderately deaf in their better ear and the remainder were severely or profoundly deaf in both ears. Overall, the mean unaided hearing levels were left ear: 90.4 db (*SD* = 20.0) and right ear: 88.7 db (*SD* = 20.1). Sixty‐nine (64%) children wore hearing aids all or some of the time, and 42 (39%) children had cochlear implants. Of the children with cochlear implants, 12 had bilateral implants, and the average age of implant was 3;2 years (*SD* = 1;9). In the deaf group as a whole, 31 used BSL as their main form of communication, 56 primarily used spoken English, and 15 were using Sign Supported English (SSE), an adapted sign system using English grammar, as their main communication mode. In order to gain a large, widely representative sample, we recruited from both specialist deaf schools (11 children from residential and 19 from nonresidential deaf schools) and mainstream educational settings (50 children from mainstream schools with specialist classrooms/units for deaf children and 28 children from mainstream schools without such resources).

In total, 125 hearing children took part in the study. The children were recruited from a wide range of primary schools in rural and urban settings, and where possible from the same school as deaf participants to control for socioeconomic status. There were 57 (46%) girls and 68 (54%) boys with a mean age of 8;11 years (*SD* = 1;5; range = 6;5–11;11). As for the deaf children, the majority of the group were White British (85%), 6 were mixed race, 7 Asian, and 6 from other backgrounds.

There were no significant differences in gender, age, or socioeconomic status (measured by parental employment status—working or not working; parent education—further education beyond compulsory schooling) between the deaf and hearing groups. Despite normal range scores for both deaf and hearing children, differences were noted in nonverbal ability and speed of processing (see below for measures) with the deaf group achieving significantly lower scores, and these are subsequently controlled for in the analyses. As a group, the deaf children also scored below 1 *SD* from the mean on vocabulary confirming that, on average, this group was language delayed.

Table 1 shows the age, gender, parental education and job status, general cognitive ability, speed of processing, and estimated standard vocabulary score of each group. For vocabulary the standard score is an estimate based on standard administration and using hearing norms. For analysis we use an adjusted raw score (see below).

**Table 1:** Descriptive Characteristics of the Sample

Group	Age	%Boys	WASI matrix *t* score^a^	Symbol search scaled score^b^	EOWPVT standardized score^c^ (unadjusted)	Parents with further education	Parents in employment
Hearing	8;11 (1;5)	54	53.0 (10.1)	12.3 (3.5)	108.18 (13.68)	79%	79%
Deaf	8;10 (1;9)	55	49.5 (10.2)	10.5 (4.4)	84.42 (18.91)	76%	73%
*p*	.544	1.00	.009	.001	< .001	.63	.86

Note.

WASI = Wechsler Abbreviated Scale of Intelligence; EOWPVT = Expressive One‐Word Picture Vocabulary Test.

^a^ *t* score norm mean is 50 (*SD* = 10).^b^Scaled score norm mean is 10 (*SD* = 3).^c^Standard score norm mean is 100 (*SD* = 15).

### Measures

#### Executive Function

*Odd one out span* (Henry, 2001) is a measure of *executive‐loaded visuospatial working memory* in which the child has to process which shape is the odd one out while storing the location of each odd shape in a grid. At the end of each trial of items, the child must recall the locations of the odd shapes in correct sequence by pointing to the correct box on a series of empty grids. Trials gradually increase in number to a maximum of six locations to recall. After two errors within a block, the test is terminated. The total number of trials with locations correctly recalled is then calculated.

In the *backward spatial span* task (Wechsler Nonverbal Scale of Ability, Wechsler & Naglieri, 2006), children are instructed to tap blocks in a sequence reversed from one shown by the experimenter. Trials gradually increase ranging up to a span of nine. After two errors at the same span length, the test is terminated and one point awarded for each correct sequence to give a score. This is also a test of *executive‐loaded visuospatial working memory*.

For *design fluency* (NEPSY, Korkman, Kirk, & Kemp, 1998), children are given a sheet of paper showing boxes containing dot arrays and instructed to produce as many different designs as possible, in 1 min, by joining two or more dots with a straight line. The assessment measures *visuospatial cognitive fluency*. A score is calculated from the total number of unique designs created.

Children's Color Trails Test 1 and 2 (Llorente, Williams, Satz, & D'Elia, 2003) is a test of *cognitive shifting*. In Test 1, children are required to draw a line connecting each numbered circle (from 1 to 15) as quickly as possible. All odd numbers are printed in a yellow circle and even numbers are printed in a pink circle. Test 2 contains two sets of encircled numbers: One set printed in a pink background and another printed in a yellow background. The child is instructed to connect numbers in ascending order, alternating between pink and yellow circles. In the present study, an interference score was calculated to give “additional time” taken in the second condition.

The Tower of London (ToL) is *an executive planning task* in which colored disks are moved from their initial position, one at a time, to match a goal set. The ToL is a simplified version of the original Tower of Hanoi task (Shallice, 1982). The Psychology Experiment Building Language version 0.14 (Mueller & Piper, 2014) was presented via laptop. Instructions were presented verbally/in sign language with use of the first trial as an example. The children completed seven remaining trials. The number of additional moves taken to complete the task over the expected number was recorded.

A computerized version of the *Simon task*, a measure of *cognitive inhibitory control*, was administered via laptop. A fixation cross appeared in the center of the screen before each trial. On each trial, a picture of a sun or an apple appeared, either left or right of center. The children were instructed to press the key marked with an apple sticker on the left‐hand side of the keyboard when they saw an apple; and when a sun picture appeared, to press the key marked with a sun sticker on the right‐hand side of the keyboard. Each stimulus appeared for 750 ms. The order of trials was randomized for each child and no feedback was given. There were a total of 32 trials, half congruent (picture on the *same* side as the response) and half incongruent (picture on the *opposite* side of the response). The increased time to respond to incongruent items is known as the Simon effect (Simon, 1990), and an “interference score” was therefore created by subtracting congruent from incongruent scores.

#### Language

The Expressive One‐Word Picture Vocabulary Test (EOWPVT; Brownell, 2000) was used to test *single‐word vocabulary production* following standard basal and ceiling administration guidelines. The children must name single pictures (primarily simple nouns, e.g., *train*; but also some verbs, e.g., *eating*, and category labels, e.g., *fruit*). The EOWPVT was developed in the United States, and so three pictures were substituted to make the test more relevant for children in the United Kingdom (e.g., *raccoon* → *badger*). Kyle et al. (2006) have previously used this measure with groups of deaf children and have predetermined acceptable signs for the items; however, in order to ensure that the EOWPVT could be used to assess the vocabulary of both hearing and signing deaf children, 15 test items that do not exist in BSL (e.g., *cactus*,* banjo*) were removed after administration. These *adjusted* EOWPVT scores are used here for fairer assessment and analysis, but using the fully scored version made no difference to any of the overall findings in this report, and the standardized means are given in Table 1 to give an indication of vocabulary level for both groups.

#### Control Tasks

The *Matrix Reasoning* subtest of the Wechsler Abbreviated Scale of Intelligence (WASI; Wechsler, 1999) was administered as a control measure for *nonverbal cognitive ability*. The child is presented with a pattern with a missing section and must select the correct response from five choices. After 4/5 successive incorrect answers, the test is terminated.

*Speed of processing* was measured using the *Symbol Search* subtest (Wechsler Intelligence Scale of Children, 3rd ed.; Wechsler, 1991). Children must identify whether the target symbol appears in rows of symbols as fast as possible.

### Procedure

Ethical approval was granted from the UCL Research Ethics Committee. Children were recruited all across the U.K. by contacting deaf schools, mainstream schools with provision for deaf children, or through the National Deaf Children's Society. Informed written consent was obtained from parents/guardians prior to testing; children gave verbal consent at the beginning of the session and were told that they could opt out at any time.

Testing took place in a quiet room at school or at the child's home. The session lasted between 60 and 75 min, and was video recorded. The children were able to take short breaks between tasks if necessary. Testing was carried out by two researchers. One was a hearing native user of BSL (i.e., an adult with deaf parents), who was highly experienced in communicating with deaf children. She used BSL in all instructions and as the main communication for testing children for whom this was the preferred language. A second experimenter, with good signing skills, tested deaf children whose preferred language was spoken English or SSE, and the hearing children. In a small number of cases where children were bilingual/bimodal (*n* = 18), the main communication mode identified by parents was not always the language chosen by the child at the point of testing. In these instances, the language chosen by the child was used as the testing language. The tasks were selected to require minimal verbal/signed instruction, and sufficient practice trials were included to ensure that the tasks were well understood. The tests were administered in the same order for all participants.

### Analysis

Simple group differences were examined using *t* tests and analysis of covariance to control for nonverbal ability and speed of processing. Correlations and partial correlations were performed using Pearson product–moment analyses. Finally, we conducted a mediation analysis following Baron and Kenny (1986), using linear regression techniques. It might be possible to use a structural equation modeling (SEM) technique with these data. However, after taking statistical advice, we concluded that with this data set (which is not longitudinal, has limited size, and has a single language measure), SEM would not add substantively to the findings and would add an element of complexity that might hinder understanding and interpretation. The fact that our hearing group is a reference sample for the EF scores also argues against the use of creating an EF factor in this way. Thus, we have opted for the simplest useful solution using regression. For each analysis some missing data are evident for specific assessments, but in all cases this was < 10% of the total cohort. Analysis was performed using SPSS v22.0. Armonk, NY: IBM Corp.

## Results

### Deaf and Hearing Group Comparisons

Deaf children scored less favorably on all of the tasks in the test battery except design fluency when compared to hearing peers. Raw vocabulary scores were also significantly different between groups, hearing group: *M *=* *86.6, *SD* = 14.1; deaf group: *M *=* *64.2, *SD* = 19.2; *t*(230) = 10.18, *p *<* *.001 *d = *1.3.

After controlling for group differences in nonverbal intelligence (as measured by WASI matrix reasoning) and speed of processing (as measured by symbol search), highly similar results were obtained. However, differences between groups on the ToL task and the color trails disappeared once nonverbal ability and speed of processing were controlled for, and design fluency remained nonsignificant (see Table 2 for details).

**Table 2:** Performance on Each Executive Function Measure (Raw Scores) by Group

Group	Odd one out score	Backward span	Design fluency score	Tower of London additional moves	Color trails additional time	Simon task interference score
Hearing	10.39 (4.46)	6.06 (1.95)	20.96 (6.35)	25.78 (15.03)	29.75 (16.90)	−11.07 (15.43)
Deaf	7.99 (4.03)	4.90 (2.11)	19.59 (7.72)	30.77 (18.16)	38.22 (20.60)	−17.03 (16.83)
	*t*(230) = −4.3 *p *< .001 *d *= .57	*t*(231) = −4.4 *p *< .001 *d *= .57	*t*(231) = −1.5 *p *= .14 *d *= .19	*t*(225) = 2.3 *p *= .025 *d *= .30	*t*(225) = 3.4 *p *= .001 *d *= .45	*t*(209) = 2.7 *p *= .008 *d *= .34

Note.

Dark gray (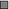) indicates differences that are significant before and after controlling for WASI (Wechsler Abbreviated Scale of Intelligence) and symbol search. Light gray (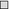) indicates differences that are significant before but not after controlling for WASI and symbol search. White (□) indicates no group differences before/after controlling for WASI and symbol search.

**Figure 1:**
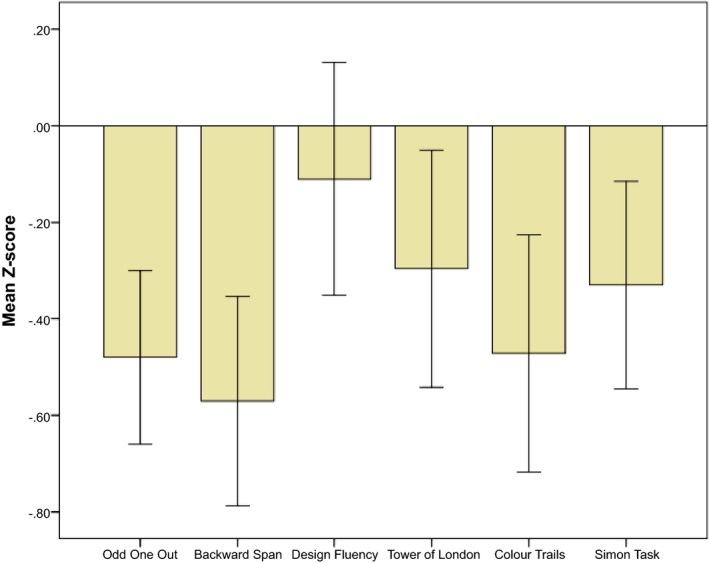
Mean *Z* score (95% CI) on each task for children in the deaf group (based on the hearing group mean *Z* = 0). [Color figure can be viewed at http://wileyonlinelibrary.com].

All EF scores were transformed into *Z* scores based on the hearing sample's mean and *SD* to allow comparison across tests and to examine how many children scored in an impaired range. Because the definition of “normal range” varies across fields, we are using a statistically based norm threshold of −1 *SD* to designate impaired scores. ToL additional moves and color trails additional time were calculated as (*Z* score × −1) to reverse scoring so that lower *Z* scores were less favorable in all cases. Figure 1 shows the pattern of the deaf group's performance across all EF tasks. Although deaf children show a disadvantage on all tasks, none of the deaf group's mean *Z* scores falls further than 1 *SD* below the mean of the hearing peers. However, a larger than expected proportion of deaf children fell into impaired ranges (−1 and −2 *SD* from the hearing group mean; see Table 3 for details). Only a small group of deaf children scored above the normal range of the hearing group (maximum *n* = 15 for Design fluency). These *Z* scores were used in all subsequent analyses and a composite EF score was created by summing these.

**Table 3:** Number (%) of Deaf Children Outside of Normal Range

Threshold	Odd one out score	Backward span	Design fluency score	Tower of London additional moves	Color trails additional time	Simon task interference score
+2 *SD*	2 (1.9)	3 (2.8)	6 (5.6)	0 (0)	0 (0)	1 (0.9)
+1 *SD*	4 (3.7)	4 (3.7)	9 (8.3)	5 (4.9)	6 (5.8)	9 (8.5)
−1 *SD*	47 (43.5%)	52 (48.1%)	28 (25.9%)	20 (19.4%)	24 (23.3%)	44 (41.5%)
−2 *SD*	2 (1.9%)	11 (10.2%)	8 (7.4%)	10 (9.7%)	12 (11.7%)	14 (13.2%)

Note.

Gray (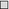) indicates differences that are significantly higher than expected from a normal distribution.

### Correlations Between EF Tasks and Language

Correlation analyses showed that EF tasks were all significantly correlated at *p *<* *.001 when the whole group (i.e., the deaf and hearing children combined) was considered (*r* values from .23 to .54) with the exception of inhibition (Simon task), which did not correlate significantly with fluency or planning (ToL). All EF tasks correlated significantly with the composite EF variable (*r* values from .4 to .77). Language was also significantly correlated with all individual measures (*r* values from .26 to .56, all *p* values < .001). Composite EF scores and vocabulary correlated strongly (*r *=* *.66, *p *<* *.001).

When groups were considered separately, the *hearing group* showed significant correlations between all EF measures (*r* values from .22 to .49), between all EF measures and the composite variable (*r* values from .36 to .75), and between EF and language (*r* values from .34 to .57) except for inhibition (Simon task), which showed no relation to any individual EF task but still showed a significant correlation with the EF composite score (*r *=* *.36, *p *<* *.001). Inhibition also showed no correlation with vocabulary (*r *=* *.14, *p *=* *.15). Composite EF scores and language correlated strongly (*r *=* *.68, *p *<* *.001).

The *deaf group* showed significant associations between all measures of EF (*r* values from .22 to .59), between all EF measures and the composite variable (*r* values from .39 to .79), and between EF and vocabulary measures (*r* values from .23 to .48). Again inhibition (Simon task) was the only exception and only showed an association with shifting (color trails; *r *=* *.22; *p *=* *.03), vocabulary (*r *=* *.23, *p *=* *.02), and the composite EF score (*r *=* *.39, *p *<* *.001). Again, composite EF scores and language correlated significantly (*r *=* *.57, *p *<* *.001; see Table 4 for within‐group EF correlations).

**Table 4:** Correlations Between Tasks for Deaf (Gray) and Hearing (White) Children

	Odd one out	Backward span	Design fluency	Color trail interference	Simon interference	Tower of London
Odd one out	1	.588	.520	.394	.105	−.360
		< .001	< .001	< .001	.285	< .001
Backward span	.464	1	.546	.363	.119	−.281
	< .001		< .001	< .001	.223	.223
Design fluency	.489	.482	1	.235	.161	−.218
	< .001	< .001		.017	.100	.015
Color trail interference	.300	.329	.251	1	.218	−.321
	.001	< .001	.005		.029	< .001
Simon interference	.122	.074	.015	.039	1	−.120
	.216	.456	.877	.695		.225
Tower of London	−.360	−.281	−.218	−.321	−.120	1
	< .001	.002	.015	< .001	.225	

Exactly the same pattern of results was seen when age was partialed out. There were two exceptions for the *deaf group* where the correlation between inhibition and switching (color trails) became nonsignificant, but the correlation between inhibition and language became significant.

### Mediation Analysis

**Figure 2:**
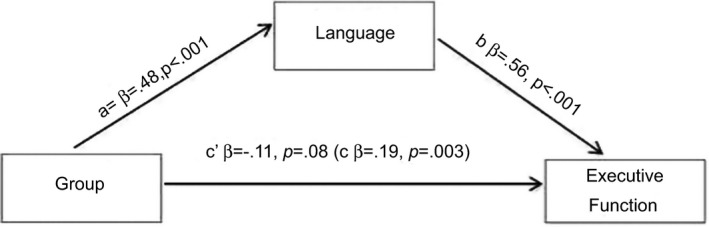
Illustration of mediation analysis a = Relation between Group and Language. b = Relation between Language and Executive Function. c = Relation between Group and Executive Function before considering Language. c′ = absence of remaining relation between Group and Executive Function once Language has been added as a mediating factor.

To test the hypothesis that language was mediating the group difference in EF scores, a series of regression analyses were completed following Baron and Kenny (1986) who state that the effect of the mediator (Language) on the dependent variable (EF) must be greater than the effect of the independent variable (Group) on the DV and that the effect of the IV (Group) on the DV (EF) should be significantly reduced or absent after controlling for the mediator (Language). This is achieved by initially running three regression analyses: (a) the direct effect of Group on Language, (b) the direct effect of Language on EF, and (c) the direct effect of Group on EF (see Figure 2). A mediation regression is then performed, examining the effect of Group (IV) on EF (DV) while controlling for Language (mediator). This is termed c′. Because groups were different on nonverbal ability and speed of processing, population norm‐based *z* scores for these variables were added in Step 1 as control variables for all regressions. For the final mediation regression, Step 2 contained the potential mediating variables (i.e., Language or EF composite *Z* score), and the final step contained the dummy variable Group (hearing/deaf).

In our sample, the direct effect of Group on EF composite (c in Figure 2) showed an adj. *R* ^2^ of .24 (β = .19; *t *=* *3.0, *p *=* *.003); the direct effect of Group on Language adj. *R* ^2^ = .39 (a in Figure 2: β = .48; *t *=* *8.9, *p *<* *.001); and the effect of Language on EF composite showed an adj. *R* ^2^ of .46 (b in Figure 2: β = .56; *t *=* *9.5, *p *<* *.001). Thus, although all models are significant, the mediator (Language) shows a substantially larger predictive value for the dependent variable (EF) than group (IV).

The effect of Group on EF after controlling for language became nonsignificant (c′ in Figure 2: β = −.11; *t *=* *−1.7, *p *=* *.08) and provided only 0.6% additional variance to the final model (adj. *R* ^2^ = .47). To confirm the direction of this effect, the reverse regression was performed exploring the effect of Group (IV) on Language (DV) after controlling for EF (mediator). In this case, Group remained a highly significant predictor of language (β = .39; *t *=* *7.9, *p *<* *.001) and added 13.4% of variance to the final model (adj. *R* ^2^ = .58).

This suggests that language is mediating group differences seen in EF performance but not vice versa. Removing Step 1 did not change the pattern of results.

## Discussion

This study aimed to provide new information about the association between language and EF by investigating these skills in deaf children, a population for whom language development is delayed by sensory rather cognitive disruption. The present research is to our knowledge the largest and most comprehensive study focusing on this population that has been conducted so far. The results of our investigation revealed two key findings.

First, even though this population presents with no primary cognitive disorder and some deaf children perform within the normal range, as a group deaf children score below hearing peers on the majority of EF tasks. The finding of lower EF in deaf children held even after accounting for speed of processing and nonverbal ability, and despite the tasks being carefully chosen for their nonverbal demands. As noted earlier, other studies have reported difficulties for deaf children on EF tasks, but these studies have important limitations. Previous results have been drawn from small groups of deaf children (Marshall et al., 2015), often only recruited from selected deaf groups such as those with cochlear implants (Kronenberger et al., 2014) or with hearing aids (Stiles et al., 2012), that cross a wider age range (Luckner & McNeill, 1994), and which have used only one or two experimental tasks or tasks that are not genuinely comparable across deaf and hearing groups (Oberg & Lukomski, 2011; Remine, Care, & Brown, 2008; Surowiecki et al., 2002). Other studies (Hauser, Lukomski, & Samar, 2013; Hintermair, 2013) have relied entirely on parent and teacher questionnaires such as the Behavior Rating Inventory of Executive Function (Gioia, Isquith, Guy, & Kenworthy, 2000), which may measure different behaviors compared to direct assessments (Jahromi, Bryce, & Swanson, 2013). Therefore, the current study confirms earlier reports of poor EF in deaf children in a larger, more representative sample using “assessment‐fair” tests of EF.

Second, our study sheds some light on whether language influences EF or whether the opposite is true. Some theorize that language is a driver in the development of EF abilities in children (e.g., Zelazo et al., 2003), whereas others describe working memory and EF as a precursor for language development (e.g., Baddeley, 2003). The results from the current study support the former hypothesis: Language not only relates to EF but also has a role in mediating EF performance. The reverse association was not evident, suggesting that poorer EF does not lead to poorer language. However, longitudinal data are needed to confirm this and at this stage our cross‐sectional data indicate only a concurrent relation.

Few studies comparing deaf and hearing children's EF have included language measures. There are three notable exceptions: Remine et al. (2008), who found no association between language and EF; Figueras et al. (2008), who assessed EF tasks and language in a fairly large sample of deaf children (*n* = 47) and found that both were lower in the deaf group, and that EF and language were highly associated. Like the findings presented here, Figueras et al. concluded that EF impairment was a result of language delay; however, their sample was not large enough to carry out a mediation analysis to investigate this further. Finally Stiles et al. (2012) noted in their small scale study (*n* = 18 deaf children) that individuals with lower working memory scores also had lower vocabulary scores. A potential limitation of our study is that we have only one measure of language skill, namely vocabulary. However, vocabulary was chosen for this study because it is one of the few ways in which the language of deaf and hearing participants can be directly compared, because the grammar of BSL is very different from that of spoken English (Sutton‐Spence and Woll, 1999). We acknowledge that no vocabulary measure will ever enable perfect cross‐language comparison between BSL and English because items will have different lexical variables in each language (e.g., frequency and age of acquisition); however, we argue that using vocabulary is the simplest available measure. Measures of receptive or productive language and syntax might reveal different relation.

Two obvious alternatives exist when considering possible reasons that language might affect EF skill. Either EF skills do not develop optimally in the context of poor language development or EF tasks (even nonverbal ones) are implicitly verbally encoded, and therefore low language skills impair performance on EF tasks. These scenarios are not mutually exclusive, and a combination of these is likely. A recent study on typically developing children and hearing children at risk of language/literacy difficulties suggested that EF and language were concurrently but not longitudinally related, which may support the latter explanation (Gooch et al., 2016). That is, deaf children's EF performance may be affected by language at the time of testing, but language may not predict later EF development. In either case, however, we are confident that the results are not a simple artifact of our carefully chosen assessment‐fair tasks.

Although this study involved a large sample of deaf participants over a wide geographical area within the UK, the vast majority of families were from a middle to high socioeconomic class. Future research is needed into atypical populations living with social disadvantage as recent work suggests that environmental factors may affect EF development (Raver, Blair, & Willoughby, 2013). Furthermore, we have not included the specific language history of deaf children within these complex analyses because subsample sizes become too small. However, when native signers have been considered in other studies, the same conclusion regarding language and cognition emerges: *Native* signers do not show the same working memory deficits as matched non‐native signers when compared to hearing peers, suggesting that rich language environment matters for EF development (Marshall et al., 2015) rather than auditory input per se as suggested by some theorists (e.g., auditory scaffolding hypothesis; Conway, Pisoni & Kronenberger, 2009). Therefore, it is not the case that all deaf children have difficulties with language and EF. Our aim in the current study was to include the whole range of deaf children so that results were not skewed by using only the most severely EF‐affected individuals.

In a wider context, the effect of language on EF may also lead to additional difficulties. Poorer EF may limit self‐regulation in everyday situations, and this has several implications for understanding the lower academic, social, and emotional behavior, and poorer impulse control of some deaf children (Beer et al., 2011; Dye & Hauser, 2014; Hauser & Marschark, 2012; McClelland et al., 2007; Stevenson, McCann, Watkin, Worsfold, & Kennedy, 2010). Further studies are needed to investigate these links directly. However, establishing language as a mediator for EF has clinical and educational implications: Language might be a useful predictor of a child's wider abilities in classroom settings; and potentially, additional early and continued language training could also boost EF performance.

Examining the ways in which atypical early language experience relates to EF performance provides us with a novel window onto possible developmental associations. Ongoing study of how language and EF are both related and separable is essential for a full understanding of both typical and atypical development, and may provide an evidence base for helping those with poorer performance in these important domains.
